# Supplementary material for: Is Sitting Always Inactive and Standing Always Active? A Simultaneous Free-Living activPal and ActiGraph Analysis
Source: Int J Environ Res Public Health. 2020 Nov 28;17(23):8864. doi: 10.3390/ijerph17238864 (PMC7730923; doi:10.3390/ijerph17238864)
Supplement: Supplementary file 1 [file ijerph-17-08864-s001.zip › Supplementary Material 1.docx]

Supplementary Material 1: Step-by-step instruction for pre-processing and behaviour classification

# Preface

The pre-processing (step 1 to 4) uses the activPal event file as a master, meaning all times and events refer to this file, and all detected times and events are written to this file. The identified valid events are, in step 4, summarized into a new variable containing all valid waking wear events that are used for the subsequent behaviour classification (step 5).

In case a different pre-processing is used, directly proceed with step 5.3c) Behaviour Classification

# Input Files

The pre-processing uses four files:

- activPal event file: format .pal or .csv, used as masterfile in step 1 to 3
- activPal raw data file: format .datx or .csv, used for sensor synchronisation in step 2
- ActiGraph 1-second count file: format .csv, used for behaviour classification in step 5
- ActiGraph raw data file: format .csv, used for sensor synchronisation in step 2 and ActiGraph non-wear detection in step 3

# 1) Identify valid activPal days

This step uses only the activPal event file.

## 1.1 Load activPal event file

1. load either .pal or .csv file to the workspace (here: MATLAB 2019a)
2. Ensure proprietary activPal codes are used (code 0 for sitting, code 1 for standing, code 2 for stepping)

## 1.2 Summarize adjacent episodes of the same activity code

1. detect adjacent events with the same activity code
2. summarize all detected events into new events (see Winkler et al. 2016)

## 1.3 Remove ultra-long events

1. detect events lasting ≥24 hours (activPal unlikely worn)
2. delete detected events

## 1.4 Stop processing if the event file contains no valid days at all

1. sum all steps and event durations among the entire file
2. remove subject if <500 steps or <12 hours (here: 5 subjects were excluded)

## 1.5 Limit data to activPal wear time

activPal wear time defined by the time in between the first/last non-sedentary event lasting 1 minute with at least 25 steps

1. detect all activPal sedentary events
2. get duration and sum steps between the sedentary events
3. find first/last non-sedentary event lasting ≥60 seconds with ≥25 steps

## 1.6 Detect bedtime

1. use the algorithm developed by Winkler et al. 2016 (here: version B)
2. mark detected bedtime with code -1

## 1.7 Adjust late bedtime start and early bedtime stop

1. get days with bedtime start after 01:00 on the next day or stop before 04:30 (here: 78 starts and 17 stops detected)
2. plot activPal classification and ActiGraph raw signal for identified days
3. mark self-reported ActiGraph waist-worn time (if available)
4. mark first/last time point with ActiGraph raw signal looking like daytime (to the nearest half an hour)
5. expand bedtime to whatever marked time occurred first (bedtime start) or last (bedtime stop) (here: 17 starts and 16 stops adjusted)

## 1.8 Remove non-valid days

1. Split data into day-by-day format by detecting events with non-equal start and stop day
2. Split detected events into the corresponding days
3. summarize for each day the number of steps, as well as the duration spent sitting (code 0), standing (code 1), and stepping (code 2)
4. delete all activPal events on days with <500 steps, <12 hours in sitting, standing and stepping, and >95% in one posture code

# 2) Synchronise raw sensor data

This step uses the activPal event file, the activPal raw data file and the ActiGraph raw data file. Note that the step is only required in case the two sensors record asynchronously. However, we so far always noticed asynchronous sensor clocks, meaning there is a time difference between the two sensors that can be approximated with a linear equation (offset = c + v × time; whereas c is the constant offset at time = 0, and v is the linear increasing offset over time). To check whether the sensor clocks are asynchronous, simply plot the raw data of both sensors against their time and inspect the curves (Figure A1)

## 2.1 Load activPal and ActiGraph raw data

1. load either the activPal .datx or .csv file and the ActiGraph .csv file to the workspace (here: MATLAB 2019a)

## 2.2 Determine the sensor clock offset

The offset is defined positive for a delayed ActiGraph raw signal, and negative for a delayed activPal raw signal.

1. linearly interpolate both sensor x-axis to the least common multiple frequency (here: 60Hz)
2. normalize interpolated data
3. detect the delay of the ActiGraph relative to the activPal in non-overlapping 3-hours episodes (limited to valid activPal data as marked in the event file in step 1) by finding the largest absolute cross-correlation, allow a maximum delay of 10 minutes (here: MATLAB function finddelay with maxlag set to 36’000 frames used), assign the delay to the middle time point of the 3-hours episode.
4. linearly approximate the detected delay over all 3-hours episodes after removing outliers (here: MATLAB function robustfit and isoutlier used)
5. visually inspect and manually adjust the delay in case the root mean squared error of the linear approximation is ≥10 seconds (here: adjusted for 1 subject)
6. apply delay to ActiGraph raw file
7. save delay to apply in step 5 to the ActiGraph 1-second count file


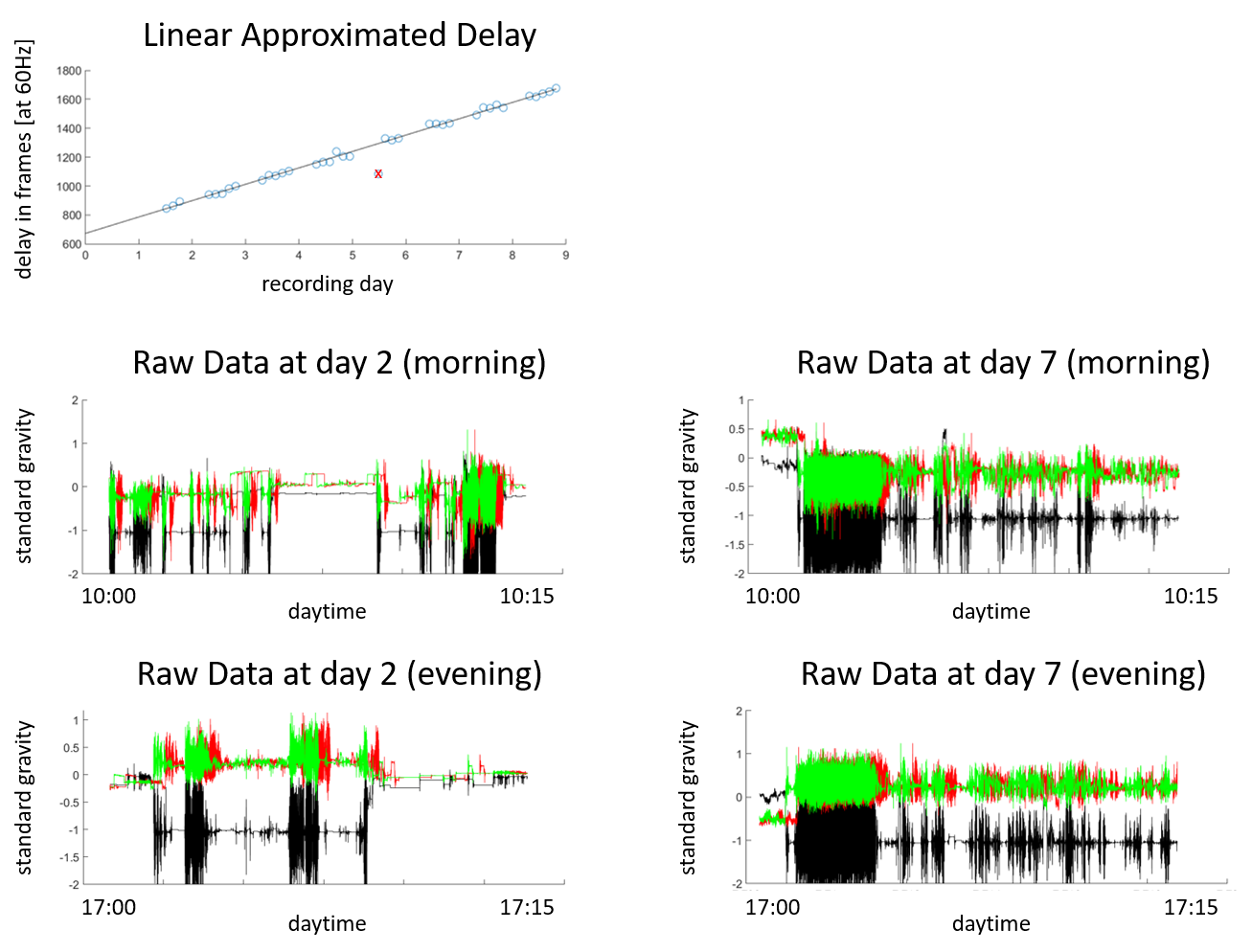


**Figure 1. Delay detection for asynchronous sensor clocks.**

The top plot shows the delay in frames at 60Hz (y-axis) for each non-overlapping 3-hour episode (blue circles) plotted at the middle time point of each episode. Note that day 0 (first recorded day) had no valid 3-hour episode, while the subsequent days had between 3 to 6 episodes. The first 3-hour episode on day 5 (marked with red cross) was detected as an outlier and thus excluded from the delay approximation. The linear approximated delay is indicated with the black line, and equal to 11.2 seconds +1.9 seconds per day (delay of ActiGraph data versus activPal data)

The remaining plots show the x-axis raw data of both sensors at selected daytimes (10:00 to 10:15, and 17:00 to 17:15) on two different days (day 2 and 7). The activPal data is shown in black, the ActiGraph data before delay correction in red and after delay correction in green. Note that the delay detection is an approximation since the two sensors were attached to different body segments and small offsets in acceleration peaks are likely (activPal on thigh, ActiGraph on waist). However, from 10:00 to 10:15 on day 2, it is very unlikely that periods of substantial acceleration changes are 16 seconds later for the waist (ActiGraph) than for the thigh (activPal), while from 17:00 to 17:15 on day 7, it is very unlikely that periods of substantial acceleration changes are 25 seconds later for the waist (ActiGraph) than for the thigh (activPal).

# 3) Exclude ActiGraph non-wear time

This step uses the activPal event file and the delay corrected ActiGraph raw data file from step 2.

## 3.1 Detect ActiGraph episodes with constant raw signal

1. get start and stop time of all ActiGraph episodes with constant raw signal on all 3 axes for ≥30 seconds

## 3.2 Mark overlapping activPal events as non-valid

1. if the activPal detects a posture change within the ActiGraph episode (criteria 1)*
2. if the activPal detects a stepping event within the ActiGraph episode (criteria 2)*
3. if the ActiGraph episode lasts for ≥90 minutes (criteria 3)

* criteria 1 and 2 neglect the first and last 5 seconds of the ActiGraph episode to take into account the non-perfect sensor clock synchronisation

1. mark detected activPal events with code -2 (ActiGraph non-wear time)

# 4) Summarize valid time and remove short episodes and short days

This step uses the activPal event file from step 3 and creates a new variable containing the start and stop time of valid waking wear time.

## 4.1 Summarize all activPal events into valid and non-valid waking wear time

Create a new variable containing the start and stop time of valid (valid-code = 1) and non-valid (valid-code = 0) waking wear time, whereas:

1. activPal events marked with code -1 (bedtime) and code -2 (ActiGraph non-wear time) are summarized as non-valid waking wear time (valid-code = 0) (note: to include bedtime in the analysis (figure 5 in manuscript), simply consider code -1 as valid wear time)
2. activPal events marked with code 0 (sitting), code 1 (standing) and code 2 (stepping) are summarized as valid waking wear time (valid-code = 1)

## 4.2 Remove short valid waking wear events

Mark valid waking wear events as non-valid if:

1. valid waking wear event is <5 minutes
2. valid waking wear event is <10 minutes and both adjacent non-valid waking wear events >2 minutes
3. valid waking wear event is <60 minutes and both adjacent non-valid waking wear events >10 minutes and together longer as the valid waking wear event

## 4.3 Remove short days

1. summarize duration of all valid waking wear events a day
2. remove days if sum is <10 hours

# 5) Behaviour Classification

This step uses the activPal event file from step 3, the ActiGraph 1-second count file (not used so far), and the new variable containing the valid waking wear time information from step 4. Note that in case both sensors were worn the entire day, valid waking wear time contains only one event for that day.

If another pre-processing was used, start the classification as outlined in section 5.3c).

## 5.1 Load and pre-process ActiGraph 1-second count file

1. Load ActiGraph 1-second count file to the workspace (here: low-frequency-extension filtered file load to MATLAB 2019a)
2. Apply ActiGraph delay saved in step 2.2 (g) to the 1-second count file

## 5.3 Classify the behaviour

The classification is done for each method separately by going through each valid waking wear event and:

1. **activPal:**
   1. extract all activPal events in the event file with the proprietary posture classification
2. **ActiGraph:**
   1. extract all 1-second ActiGraph counts lying within the valid waking wear event
   2. sum 1-second ActiGraph counts into non-overlapping 1-minute events
   3. classify each 1-minute event with the predefined counts-per-minute cut-points (here: 100 cpm and 1’952 cpm) into minimal-intensity physical activity, light-intensity physical activity, and moderate- to vigorous-intensity physical activity*
   4. summarize adjacent minutes with the same classification into bouts

*Note that the last seconds of each valid waking wear event not summing to 1 minute are classified with the corresponding fraction of the counts-per-minute cut-point

1. **POPAI:**
   1. Extract all activPal events in the event file with the proprietary posture classification
   2. Go through each activPal sitting and standing event and
      1. extract all 1-second ActiGraph counts lying within the activPal event
      2. sum 1-second ActiGraph counts into non-overlapping 1-minute events
      3. classify each 1-minute event with the predefined posture-specific counts-per-minute cut-points (here: 75 cpm for sitting and 150 cpm for standing) into inactive (minimal-intensity physical activity) and active (light-intensity or moderate- to vigorous-intensity physical activity) sitting and standing*
      4. summarize adjacent minutes with the same classification into longer events

* Note that activPal events <1 minute and the last second of each longer activPal event not summing to 1 minute (e.g. the last 56.3 seconds of a 116.3 second event) are classified with the corresponding fraction of the counts-per-minute cut-point
