# Supplementary material for: Is Sitting Always Inactive and Standing Always Active? A Simultaneous Free-Living activPal and ActiGraph Analysis
Source: Int J Environ Res Public Health. 2020 Nov 28;17(23):8864. doi: 10.3390/ijerph17238864 (PMC7730923; doi:10.3390/ijerph17238864)
Supplement: Supplementary file 1 [file ijerph-17-08864-s001.zip › Supplementary Material 2.docx]

Supplementary Material 2

**Figure 1. 24-hour behaviour pattern**


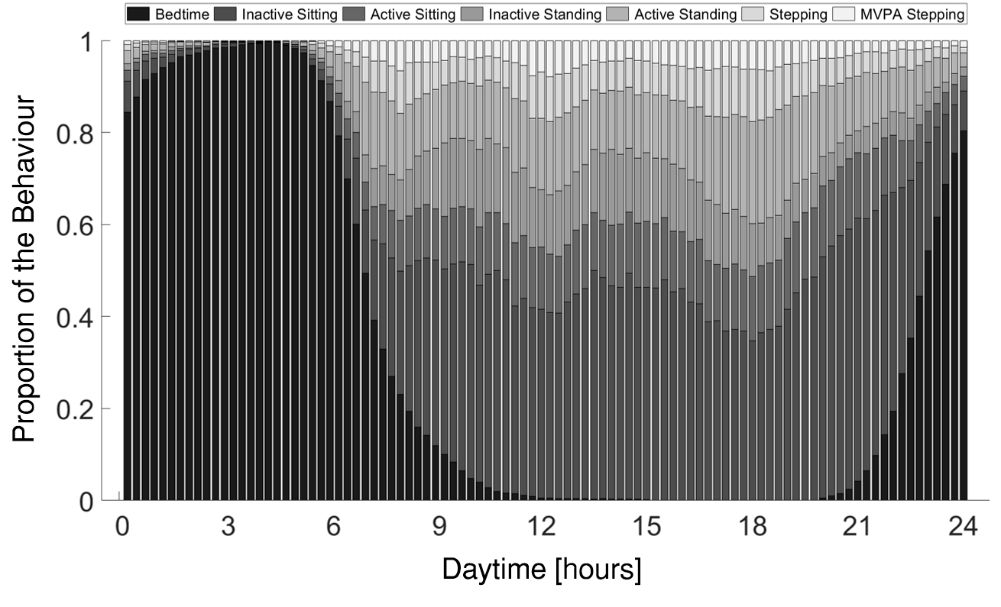


Example of an advanced posture and physical activity classification with POPAI. The figure shows the proportion of each behaviour (y-axis) over the whole day (x-axis, each bar represents 15 minutes). The behaviour classification includes bedtime (detected with an activPal algorithm, see Ref 20 of main manuscript) and separates MVPA stepping from normal stepping (using a vertical axis cut-point of 1’952 cpm). Detailed instructions on how to classify the behaviour are included in Supplementary Material 1.
